# Loss of leucine-rich repeat kinase 2 causes age-dependent bi-phasic alterations of the autophagy pathway

**DOI:** 10.1186/1750-1326-7-2

**Published:** 2012-01-09

**Authors:** Youren Tong, Emilie Giaime, Hiroo Yamaguchi, Takaharu Ichimura, Yumin Liu, Huiqing Si, Huaibin Cai, Joseph V Bonventre, Jie Shen

**Affiliations:** 1 Center for Neurologic Diseases, Department of Neurology, Brigham and Women's Hospital, Program in Neuroscience, Harvard Medical School, Boston, MA 02115, USA; 2 Renal Division, Department of Medicine, Brigham and Women's Hospital, Harvard Medical School, Boston, MA 02115, USA; 3 Unit of Transgenesis, Laboratory of Neurogenetics, National Institute on Aging, National Institutes of Health, Bethesda, MD 20892, USA

**Keywords:** LRRK2, Parkinson's disease, knockout, LC3, p62, lysosomal proteins, cathepsins, lipofuscin

## Abstract

**Background:**

Dominantly inherited missense mutations in leucine-rich repeat kinase 2 (LRRK2) are the most common genetic cause of Parkinson's disease, but its normal physiological function remains unclear. We previously reported that loss of LRRK2 causes impairment of protein degradation pathways as well as increases of apoptotic cell death and inflammatory responses in the kidney of aged mice.

**Results:**

Our analysis of *LRRK2*-/- kidneys at multiple ages, such as 1, 4, 7, and 20 months, revealed unique age-dependent development of a variety of molecular, cellular, and ultrastructural changes. Gross morphological abnormalities of the kidney, including altered size, weight, texture, and color, are evident in *LRRK2*-/- mice at 3-4 months of age, along with increased accumulation of autofluorescent granules in proximal renal tubules. The ratio of kidney/body weight in *LRRK2*-/- mice is increased at 1, 4, and 7 months of age (~10% at 1 month, and ~20% at 4 and 7 months), whereas the ratio is drastically decreased at 20 months of age (~50%). While kidney filtration function evaluated by levels of blood urea nitrogen and serum creatinine is not significantly affected in *LRRK2*-/- mice at 12-14 months of age, expression of kidney injury molecule-1, a sensitive and specific biomarker for epithelial cell injury of proximal renal tubules, is up-regulated (~10-fold). Surprisingly, loss of LRRK2 causes age-dependent bi-phasic alterations of the autophagic activity in *LRRK2*-/- kidneys, which is unchanged at 1 month of age, enhanced at 7 months but reduced at 20 months, as evidenced by corresponding changes in the levels of LC3-I/II, a reliable autophagy marker, and p62, an autophagy substrate. Levels of α-synuclein and protein carbonyls, a general oxidative damage marker, are also decreased in *LRRK2*-/- kidneys at 7 months of age but increased at 20 months. Interestingly, the age-dependent bi-phasic alterations in autophagic activity in *LRRK2*-/- kidneys is accompanied by increased levels of lysosomal proteins and proteases at 1, 7, and 20 months of age as well as progressive accumulation of autolysosomes and lipofuscin granules at 4, 7-10, and 20 months of age.

**Conclusions:**

LRRK2 is important for the dynamic regulation of autophagy function *in vivo*.

## Background

Parkinson's disease (PD) is the most common neurodegenerative movement disorder. The neuropathological hallmarks of PD are progressive degeneration of dopaminergic neurons in the *substantia nigra pars compacta *of the brain and the presence of intraneuronal cytoplasmic inclusions known as Lewy bodies (LBs), in which α-synuclein aggregates are a major component [1,2]. Although most PD cases occur sporadically, at least five genes (including *α-synuclein*, *parkin*, *DJ-1*, *PINK1*, and *LRRK2*) associated with monogenetic familial forms of the disease mimicking clinical symptoms of sporadic PD have been identified, permitting studies of the pathogenic mechanisms of PD using genetic approaches. Dominantly inherited missense mutations in the *leucine-rich repeat kinase 2 *(*LRRK2*) gene are the most common genetic cause of late-onset PD [3-9], highlighting the importance of LRRK2 in PD pathogenesis. LRRK2 is a large protein of 2527 amino acid residues, consisting of several functional domains, including a Ras-like small GTPase domain, a MAP kinase-like domain, as well as several protein-protein interaction domains, such as the leucine-rich repeat domain [6,9,10]. The disease-associated mutations in LRRK2 are present in all functional domains of the protein. Most LRRK2 mutations causes clinically typical PD, but the neuropathological features vary, ranging from pure nigral degeneration without LBs to nigral degeneration with brainstem or widespread LBs, or ubiquitin-positive inclusions, or neurofibrillary tau-positive tangles [9,11].

Despite the disease relevance of LRRK2, its normal physiological role remains elusive. Elucidation of LRRK2 functions will provide insights into how mutations in LRRK2 lead to dopaminergic dysfunction and degeneration. Although the dominant inheritance of missense mutations and the lack of nonsense or deletion mutations in *LRRK2 *are consistent with toxic gain-of-function pathogenic mechanisms, we generated *LRRK2*-/- mouse models to study the normal physiological function of LRRK2 and to determine the consequence of inhibiting LRRK2 function. Similar to other PD genetic mouse models, such as α-synuclein transgenic [12-14], *parkin*-/- [15,16], *DJ-1*-/- [17,18], *PINK1*-/- [19,20], and *LRRK2 *transgenic and knockin mice [21-25], *LRRK2*-/- brains did not develop overt dopaminergic degeneration [26]. However, *LRRK2*-/- kidneys developed striking age-dependent abnormalities that are relevant to PD pathogenesis, such as impairment of protein degradation pathways, apoptotic cell death, oxidative damage, and inflammatory responses [26]. There was striking accumulation and aggregation of α-synuclein and ubiquitinated proteins in the kidneys of *LRRK2*-/- mice at 20 months of age [26]. The autophagy-lysosomal pathway, which has been implicated in various neurodegenerative diseases with protein aggregation-related pathologies, including Parkinson's disease and Huntington's disease [27-30], was impaired in *LRRK2*-/- kidneys at 20 months of age, as indicated by impaired conversion of LC3-I to LC3-II, a reliable indicator of the autophagic activity [31], and accumulation of p62, an autophagy substrate [32]. Although these molecular and cellular changes are observed only in the kidney but not in the brain of *LRRK2*-/- mice, they are very similar to processes that are thought to be involved in PD pathogenesis, making *LRRK2*-/- kidneys a relevant and valuable *in vivo *model to study the physiological function of LRRK2 and to identify the downstream cellular and molecular pathways.

In the current study, our detailed time course study revealed an unexpected finding that loss of LRRK2 dysregulates the autophagy pathway in an age-dependent bi-phasic manner. The autophagic activity is elevated at young ages (e.g., 7 months) but reduced at an old age (e.g., 20 months). Furthermore, this process is accompanied by increased levels of lysosomal proteins and proteases as well as age-dependent, progressive accumulation of autolysosomes and lipofuscin granules. Thus, subsequent impairment of autophagy function in aged *LRRK2*-/- kidneys may be due to depletion of autophagy machinery and accumulation of subcellular structures containing undigested lysosomal components during aging.

## Results

### Morphological and histological analyses of *LRRK2*-/- kidneys at various ages

**Figure 1:**
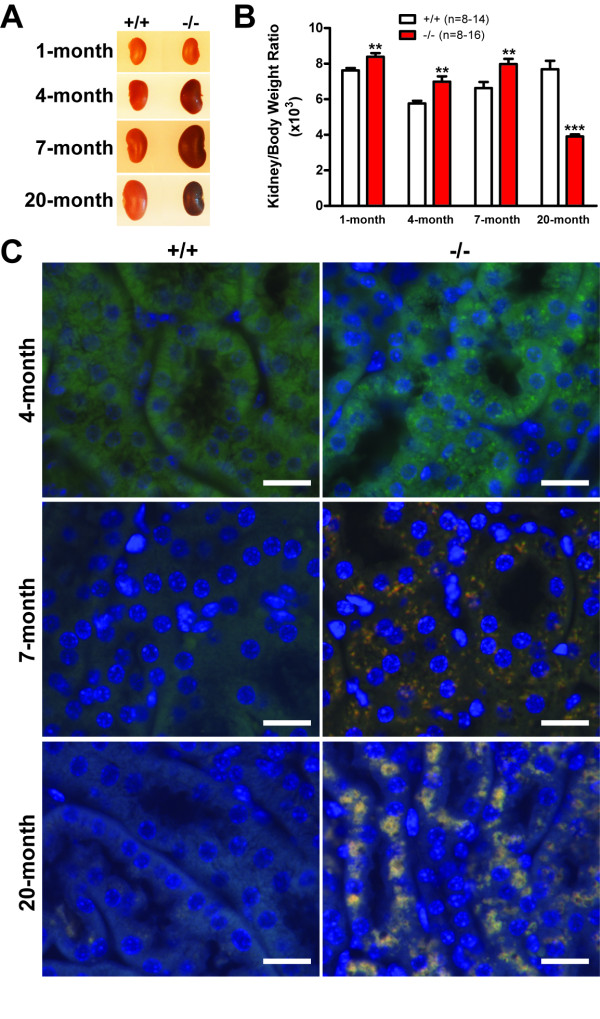
**Morphological and histological analyses of *LRRK2*-/- kidneys at various ages**. **A**. Initial discoloration, enlarged size, and altered granular tissue texture are observed in kidneys of male *LRRK2*-/- mice beginning at 3-4 months of age, the extent of abnormalities increases with age. **B**. The ratio of kidney/body weight is significantly increased in male *LRRK2*-/- mice at the ages of 1, 4, and 7 months but decreased drastically at 20 months of age. **C**. Autofluorescence in the epithelial cells of the proximal tubules of the renal cortex of *LRRK2*-/- mice. Autofluorescent intracellular granules are more visible in the epithelial cells of the proximal tubules of *LRRK2*-/- kidneys at 7 months than 4 months of age. By 20 months of age, the cytosolic regions of the epithelial cells of the proximal tubules in the *LRRK2*-/- kidneys are occupied by large but irregular structures showing strong autofluorescence. DAPI-stained nuclei are in blue. All scale bars: 20 μm.

We recently reported that while *LRRK2*-/- mice did not develop overt dopaminergic degeneration and neuropathological changes in the brain up to two years of age, loss of LRRK2 caused striking abnormalities in the kidney, which normally expresses the highest level of LRRK2 compared to other organs and tissues [26]. To determine when *LRRK2*-/- kidneys first show morphological changes, we performed age-dependent analysis of *LRRK2*-/- mice. Because *LRRK2*-/- kidneys appear grossly normal at 10 weeks of age [26], we performed additional analysis at 4 and 7 months of age, and found that initial discoloration and altered granular tissue texture became evident in the kidney of *LRRK2*-/- mice at 3-4 months of age (Figure 1A). Interestingly, the weight and size of *LRRK2*-/- kidneys undergo bi-phasic changes as the mice get older, with initial increases in weight and size followed by subsequent drastic decreases at 20 months of age (Figure 1B). The male *LRRK2*-/- kidneys appear to have more severe morphological abnormalities (darker color and rougher surfaces), whereas female mice exhibit more variation in the kidney weight and size. We therefore used only male mice in all of the subsequent analyses. In contrast to *LRRK2*-/- mice at 20 months of age, which show ~49% decrease in the ratio of kidney/body weight compared with wild-type controls [26], kidneys from *LRRK2*-/- mice at 1, 4, and 7 months of age are larger in size and weigh more compared with wild-type controls (~10% increase at 1 month and ~20% increase at 4 and 7 months in the ratio of kidney/body weight) (Figure 1B).

In addition to the gross morphological abnormalities in *LRRK2*-/- kidneys beginning at 3-4 months of age, we observed many small autofluorescent puncta in the epithelial cells of the proximal tubules in the deep layer of the renal cortex in *LRRK2*-/- mice at 4 months of age, which became more evident and distributed more widely at 7 months of age (Figure 1C). By 20 months of age, the cytosolic regions of the epithelial cells of the proximal renal tubules are filled with larger autofluorescent structures (Figure 1C) that are lipofuscin granules [26]. These data suggest that undigested autofluorescent materials accumulate in *LRRK2*-/- kidneys beginning as early as 4 months of age.

### Up-regulation of kidney injury molecule-1 (Kim-1) in *LRRK2*-/- kidneys

**Figure 2:**
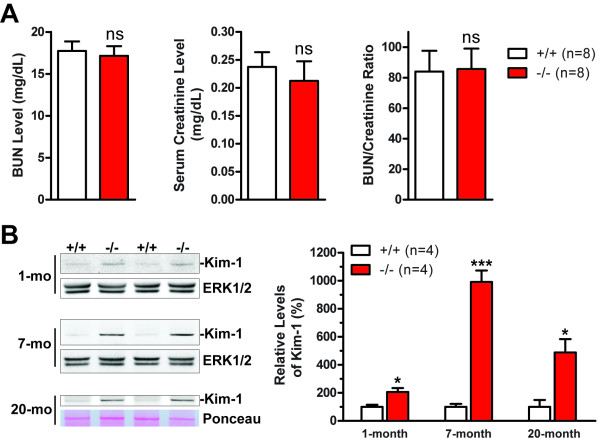
**Kidney injury and functional assay of *LRRK2*-/- mice**. **A**. Classical renal chemistry analysis shows normal levels of blood urea nitrogen (BUN) and serum creatinine as well as the ratio of BUN to serum creatinine in *LRRK2*-/- mice at 12-14 months of age. **B**. Western analysis shows dramatically increased levels of kidney injury molecule-1 (Kim-1), a tissue and urinary biomarker for renal injury, in RIPA buffer-soluble fractions of *LRRK2*-/- kidneys at the ages of 1 and 7 months, which is also increased in RIPA buffer-insoluble fractions of *LRRK2*-/- kidneys at 20 months of age, compared with wild-type controls. The Western blots of ERK1/2 or Ponceau S staining of the membranes are used as loading control, as levels of both GAPDH and β-actin are altered in *LRRK2*-/- kidneys. *ns*, not significant; *, *P *< 0.05; ***, *P *< 0.001.

We also assessed whether accumulation and aggregation of proteins in the kidney caused any loss of renal function by measuring the levels of blood urea nitrogen (BUN) and serum creatinine, a classical method of assessing renal function [33]. There is no significant difference in the levels of BUN and serum creatinine between *LRRK2*-/- mice and wild-type controls at 12-14 months of age (Figure 2A). The BUN-to-creatinine ratio, which is used to determine the possible cause of acute kidney injury, is also normal in *LRRK2*-/- mice (Figure 2A), suggesting that the renal filtration function is not significantly affected in *LRRK2*-/- mice up to 12-14 months of age.

To assess whether the striking abnormalities observed in *LRRK2*-/- kidneys caused any nephrotoxicity to epithelial cells of proximal renal tubules, we examined the levels of kidney injury molecule-1 (Kim-1 in rodents, KIM-1 in human), a very sensitive and specific biomarker for epithelial cell injury of proximal renal tubules in various settings [33,34]. An increase in Kim-1 due to kidney injury can occur before any significant increase in serum creatinine [34]. Kim-1 is localized to proximal tubule epithelial cells, and its expression is at a very low level in normal kidneys but increases dramatically after acute kidney injury [34]. Long-term expression of KIM-1 is also observed in patients with chronic kidney disease although its level is lower than that after acute kidney injury [33]. There was already a significant increase in the level of Kim-1 in *LRRK2*-/- kidneys at one month of age (Figure 2B), which then appeared grossly normal. At 7 months of age, there was approximately 10-fold increase in the level of Kim-1 in *LRRK2*-/- kidneys compared with wild-type controls (Figure 2B), but this increase was much lower than that in acute kidney injury models (> 500-fold up-regulation), such as those induced by ischemia [34]. The increased expression of Kim-1 in *LRRK2*-/- kidneys persisted to 20 months of age (Figure 2B). These data suggest that while renal function evaluated by measuring blood urea nitrogen and serum creatinine appears normal, *LRRK2*-/- mice sustain chronic kidney injury, as indicated by 10-fold up-regulation of kidney injury molecule-1.

### Age-dependent bi-phasic alterations of autophagic activity in *LRRK2*-/- mice

**Figure 3:**
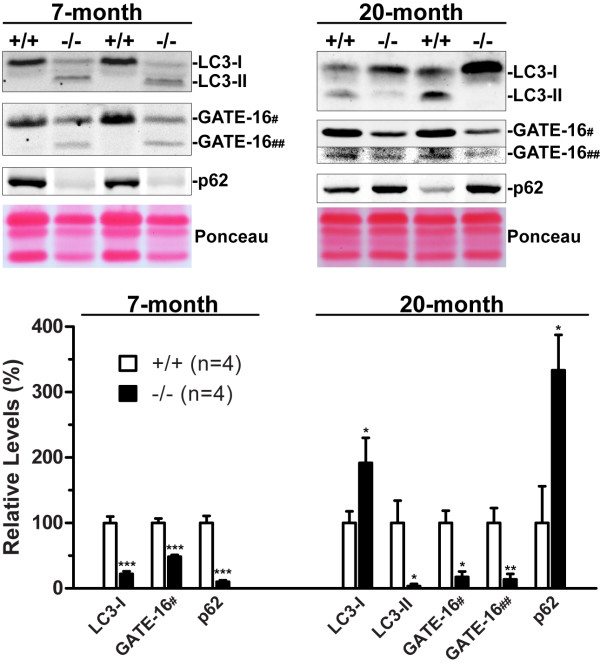
**Loss of LRRK2 causes age-dependent bi-phasic alterations of autophagy markers or substrates**. Western analysis shows bi-phasic alterations of proteins (LC3-I and -II, GATE-16 (#, form I; ##, form II)) involved in the autophagy-lysosomal pathway as well as autophagy substrate (p62) in the kidney of *LRRK2*-/- mice at the ages of 7 and 20 months compared with their respective wild-type (+/+) controls. Western blots shown were obtained using RIPA buffer-insoluble fractions. The overall contrast and brightness for the GATE-16## panel were adjusted to make the band more visible, since the level of GATE-16 form II is much lower than that of GATE-16 form I. In the bar-graph showing quantification of proteins, the data for LC3-II and form II of GATE-16 from 7 months of age are not included, as these proteins are not detectable in WT controls under these conditions. *, *P *< 0.05; **, *P *< 0.01; ***, *P *< 0.001.

To better understand the molecular mechanism underlying age-dependent protein accumulation and aggregation in the kidney of *LRRK2*-/- mice, we further investigated the effect of LRRK2 deletion on the autophagy-lysosomal pathway, one of the major protein degradation pathways. Autophagy is often referred to as macroautophagy, the major type of autophagy, by which long-lived or damaged proteins and organelles together with part of the cytoplasm are first enclosed by double-membrane structures to form autophagosomes, which then fuse with lysosomes to form autolysosomes and the cargo delivered by autophagosome gets degraded by lysosomal acid hydrolases and recycled back to the cytoplasm [27-30,35]. We previously reported that the autophagy-lysosomal pathway was impaired in *LRRK2*-/- kidneys at 20 months of age [26], as indicated by accumulation of lipofuscin granules as well as impaired conversion of non-lipidated form (LC3-I) to lipidated form (LC3-II) of microtubule-associated protein 1 light chain 3 (LC3), a reliable indicator for autophagosome formation [31], and accumulation of p62, an autophagy substrate [32]. Surprisingly, Western analysis showed higher levels of LC3-II and lower levels of LC3-I in *LRRK2*-/- kidneys at 7 months of age, as well as lower levels of p62 (Figure 3). There were no significant alterations in the levels of LC3 and p62 in the brain of *LRRK2*-/- mice compared to wild-type controls at 20 months of age (data not shown). These data suggest the increased conversion of LC3-I to LC3-II and enhanced autophagic activity (i.e., increased protein degradation) in the *LRRK2*-/- kidneys at 7 months of age, which were opposite to those at 20 months of age (Figure 3).

Western blotting also revealed an increased conversion of form I to form II of Golgi-associated ATPase enhancer of 16 kDa (GATE-16), which is a homolog of LC3 and has also been reported to localize to autophagosomal membrane upon form-II formation [36], in *LRRK2*-/- kidneys at 7 months of age, further confirming enhanced autophagic activity. By 20 months of age, both forms I and II of GATE-16 were decreased in kidneys of *LRRK2*-/- mice (Figure 3). These results indicate that loss of LRRK2 *in vivo *increases autophagic activity initially followed by subsequent decreases of autophagic activity.

### Age-dependent bi-phasic alterations of α-synuclein levels in *LRRK2*-/- kidneys

**Figure 4:**
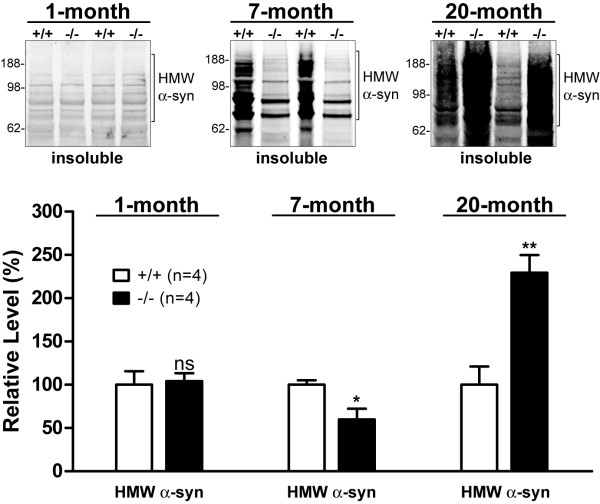
**Loss of LRRK2 causes age-dependent bi-phasic alterations of α-synuclein**. Representative Western blots show that while there is no significant difference in the levels of α-synuclein between *LRRK2*-/- mice and wild-type (+/+) controls at one month of age, high-molecular-weight (HMW) α-synuclein-immunoreactivity is significantly decreased in the RIPA buffer-insoluble fractions of *LRRK2*-/- kidneys at 7 months of age, suggesting increased degradation of α-synuclein. The level of HMW α-synuclein immunoreactivity is increased dramatically in the RIPA buffer-insoluble fractions of *LRRK2*-/- kidneys at 20 months of age, suggesting increased aggregation of α-synuclein. The specificity of the α-synuclein antibody had been confirmed previously using samples from *α-synuclein*-/- mice [37] and were confirmed again using samples from multiple independent lines of α-synuclein-overexpressing transgenic mice [14,23]. *ns*, not significant; *, *P *< 0.05; **, *P *< 0.01.

α-Synuclein has been reported to be degraded at least in part through the autophagy-lysosomal pathway, and especially the clearance of α-synuclein aggregates is highly dependent on the autophagy-lysosomal pathway [27,30]. We therefore measured levels of α-synuclein in both soluble and insoluble fractions of *LRRK2*-/- and control kidneys at the ages of 1, 7, and 20 months by Western blotting using a specific α-synuclein antibody, which had been tested previously using samples from *α-synuclein*-/- mice [37] and from transgenic mice overexpressing α-synuclein [23]. We found that while at the ages of 1 and 7 months there was little α-synuclein that was detectable by Western blotting in the RIPA buffer-soluble fraction of the kidneys of both *LRRK2*-/- mice and wild-type controls (data not shown), the levels of high-molecular-weight species that were immunoreactive for α-synuclein were reduced by approximately 40% in the RIPA buffer-insoluble fractions of *LRRK2*-/- kidneys at 7 months of age compared with wild-type controls, though no difference was found between the genotypes at 1 month of age (Figure 4). By 20 months of age, there were huge accumulation (~60-fold) of α-synuclein in the RIPA buffer-soluble fractions and significant increases (~1-fold) of high-molecular-weight α-synuclein-immunoreactive species in the RIPA buffer-insoluble fractions of *LRRK2*-/- kidneys (Figure 4) [26]. Thus, levels of α-synuclein were normal in *LRRK2*-/- kidneys at 1 month of age, decreased at 7 months, and increased at 20 months. These results are consistent with other markers of autophagy function (Figure 3) and indicate that autophagic activity is enhanced in *LRRK2*-/- kidneys at 7 months of age but impaired by 20 months of age.

### Age-dependent bi-phasic alterations of oxidation levels in *LRRK2*-/- kidneys

**Figure 5:**
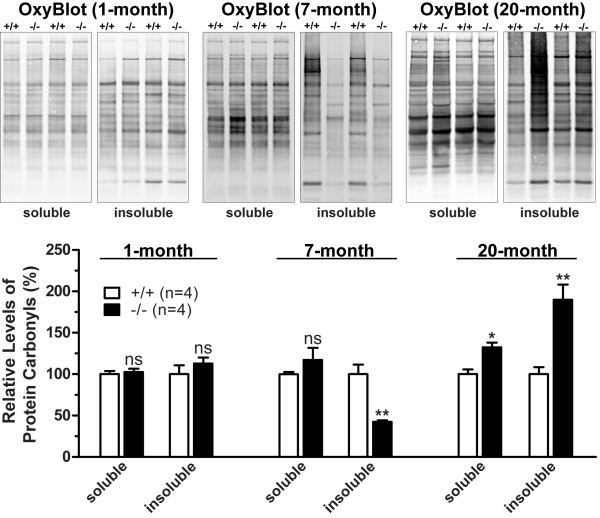
**Loss of LRRK2 causes age-dependent bi-phasic alterations of protein carbonyls**. There is no significant difference in the levels of protein carbonyls, determined using OxyBlots, in both RIPA buffer-soluble and -insoluble fractions of kidneys between *LRRK2*-/- mice and wild-type (+/+) controls at one month of age. But the level of protein carbonyls is significantly reduced in the RIPA buffer-insoluble fractions of *LRRK2*-/- kidneys at 7 months of age compared with wild-type controls. By 20 months of age, the level of protein carbonyls is significantly increased in both RIPA buffer-soluble and -insoluble fractions of *LRRK2*-/- kidneys. *ns*, not significant; **, *P *< 0.01.

Autophagy can be regulated by oxidative stress and oxidized proteins are degraded *via *the autophagy-lysosomal pathway [28,38-40]. The levels of protein carbonyls, a general marker of oxidative damage, was dramatically increased in the kidneys of *LRRK2*-/- mice at 20 months of age [26], consistent with abnormal accumulation of lipofuscin granules, which are composed of undigested materials after lysosomal degradation containing oxidized lipids, carbohydrates, and proteins, and are undegraded aggregates as a result of excessive oxidation and crosslinking [38]. However, *LRRK2*-/- kidneys at 7 months of age showed a decreased oxidation level, indicated by the reduced levels of protein carbonyls in the RIPA buffer-insoluble fractions of the kidneys (Figure 5). There was no significant difference in the levels of protein carbonyls in both RIPA buffer-soluble and -insoluble fractions of *LRRK2*-/- kidneys at one month of age (Figure 5). These results are consistent with increased intracellular degradation of oxidized proteins due to enhanced autophagic activity in *LRRK2*-/- kidneys at 7 months of age.

### Accumulation of lysosomal proteins/proteases and autolysosomes in *LRRK2*-/- mice

**Figure 6:**
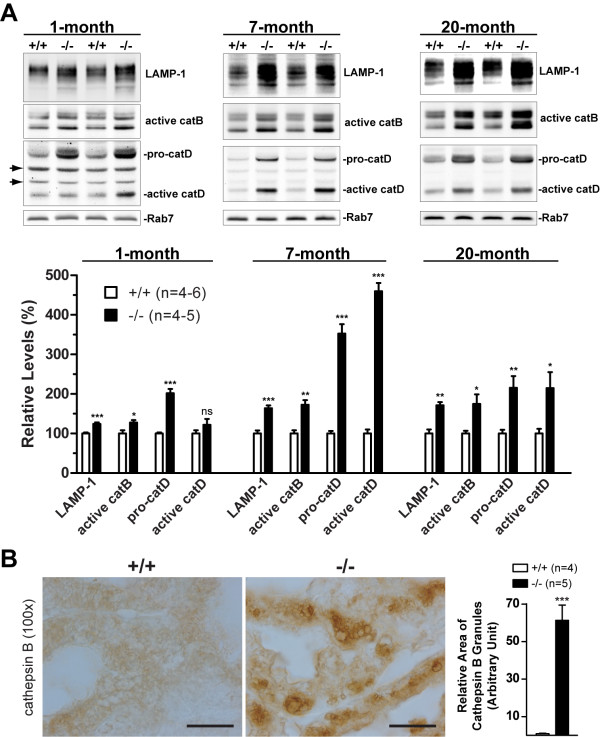
**Loss of LRRK2 causes accumulation of lysosomal proteins and proteases at all ages examined**. **A**. Representative Western blots show accumulated levels of lysosomal-associated membrane protein (LAMP-1) and active forms (cathepsins B (catB) and D (catD)) and proforms (cathepsin D (pro-catD)) of lysosomal proteases involved in the autophagy-lysosomal pathway in the kidneys of 1-, 7-, and 20-month-old *LRRK2*-/- mice. All the blots shown here were obtained using RIPA buffer-soluble fractions. Arrows indicate non-specific bands. The Western blots of Rab7 are used as loading control. *ns*, not significant; *, *P *< 0.05; **, *P *< 0.01; ***, *P *< 0.001. **B**. Immunohistochemical analysis revealed increased immunoreactivity of cathepsin B in the *LRRK2*-/- kidney at 20 months of age, which is associated with or clustered at granular structures. All scale bars: 20 μm.

Autophagy and lysosomes are closely linked in their involvement in degradation of damaged molecules and organelles [28,31,41,42]. We therefore measured levels of lysosomal proteins and proteases in *LRRK2*-/- kidneys at 1, 7, and 20 months of age (Figure 6). Western blotting analysis showed increased levels of lysosomal-associated membrane proteins LAMP-1 (Figure 6A) and LAMP-2 (data not shown) in the kidneys of *LRRK2*-/- mice at 1, 7, and 20 months of age. Levels of lysosomal proteases cathepsins B and D (both proforms and active forms) are also elevated in *LRRK2*-/- kidneys (Figure 6A). Immunohistochemical analysis showed increased immunoreactivity of cathepsin B in *LRRK2*-/- kidneys at both 7 and 20 months of age, which appeared mostly clustered at granular structures (Figure 6B).

**Figure 7:**
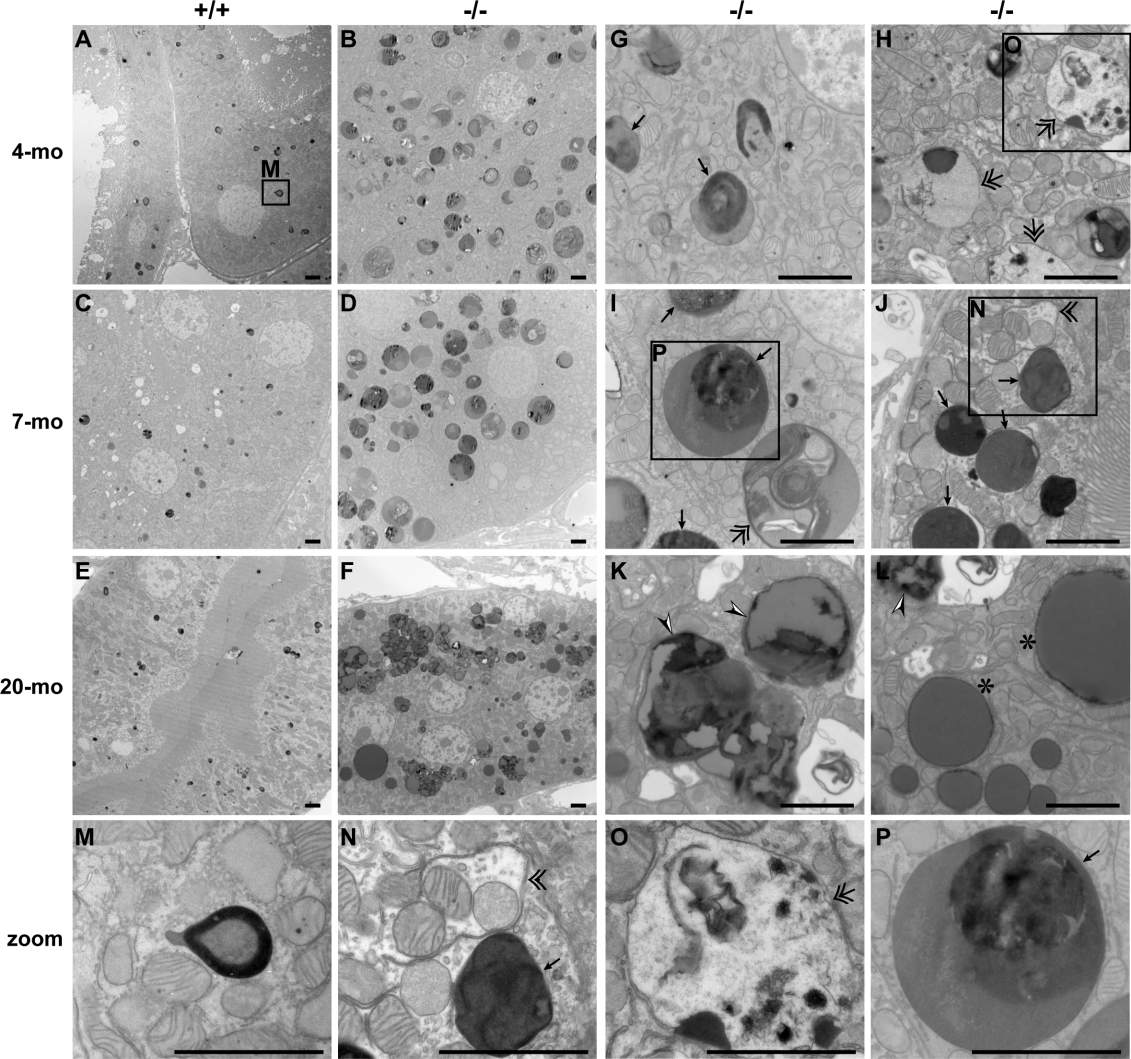
**Electron microscopy analysis of kidneys from *LRRK2*-/- mice**. EM analysis shows the presence of many electron-dense but heterogeneous autophagic vacuoles (autophagosomes (double arrows), autolysosomes (single arrows)) at 4 months of age (**A**, **B**, **G**, **H**), striking accumulation of giant electron-dense autolysosomes-like structures (single arrows) at 7 months of age (**C**, **D**, **I**, **J**), and typical tripartite lipofuscin granules (arrow heads) as well as round lipid vacuoles (asterisks) at 20 months of age (**E**, **F**, **K**, **L**) in the epithelial cells of the proximal tubules (see the characteristic brush-like tall microvilli) in the cortical area of the kidneys of *LRRK2*-/- mice. The images of the bottom row (**M**, **N**, **O**, **P**) are higher-magnification views of boxed areas above, showing a lysosome (**M**), a being-formed autophagosome (double arrowhead; **N**), a double membrane-bound autophagosome (double arrow; **O**), and an autolysosome with an electron dense core (single arrow; **P**), respectively. All scale bars: 2 μm.

We further performed electron microscopy analysis of *LRRK2*-/- and wild-type kidneys at the ages of 4, 7, 9-10, and 20 months, and found age-dependent accumulation of electron-dense autolysosomes in the epithelial cells of proximal tubules of *LRRK2*-/- kidneys (Figure 7). Autolysosome is an organelle derived from the fusion of an autophagosome and a lysosome, and is where proteins and organelles are digested [28,31,41,42]. At 4 months of age, the presence of a large number of electron-dense autophagosome-like structures as well as autolysosome-like structures was already evident in *LRRK2*-/- kidneys and such structures were absent in wild-type kidneys (Figure 7A, B, G, &7H). At the ages of 7 months (Figure 7C, D, I, &7J) and 9-10 months (data not shown), autophagosome-like structure as well as autophagic vacuoles that were being formed and engulfing organelles (e.g., mitochondria) were also present in *LRRK2*-/- kidneys, consistent with the enhanced autophagic activity at 7 months of age (Figure 3). However, autolysosome-like structures in the kidneys of 7-month-old *LRRK2*-/- mice were larger and more abundant than those at 4 months of age. By 20 months of age, we observed in *LRRK2*-/- kidneys very large to huge electron-dense lipofuscin granules of typical tripartite structure composed of three morphologically recognizable components, i.e., irregular electron-lucid component, lipid component of intermediate electron-density, and electron-dense component containing ferritin-like grains [43], and largely round lipid vacuoles (Figure 7F, K, &7L). These granules are very different from the electron-dense autolysosome-like structures that are abundant in the kidney of *LRRK2*-/- mice at the ages of 7 months and 9-10 months. Occasionally, some smaller lipofuscin-like granules were observed in *LRRK2*-/- kidneys at 7 and 9-10 months of age (data not shown). These autolysosomes and lipofuscin granules may be the sources of the strong autofluorescence observed in *LRRK2*-/- kidneys (Figure 1C). In addition, normal lysosomes were barely observed in *LRRK2*-/- kidneys at 7, 9-10, and 20 months of age. Our EM analysis of brain samples from *LRRK2*-/- mice did not show abnormal accumulation of autophagosomes, autolysosomes, and lipofuscin granules, consistent with our previous report demonstrating the absence of overt neuropathological changes [26]. Together these results show that loss of LRRK2 results in accumulation of lysosomal proteins and proteases as well as autolysosomes, which eventually develop into lipofuscin granules (and impair autophagy function) in aged kidneys.

## Discussion

Dominantly inherited mutations in LRRK2 are collectively the most common genetic cause of PD, but its normal physiological function remains less clear. We reported previously that loss of LRRK2 causes impairment of the two major protein degradation pathways (i.e., the autophagy-lysosomal pathway and the ubiquitin-proteasome system), accumulation and aggregation of proteins (such as α-synuclein, p62, and ubiquitinated proteins), and increased apoptotic cell death and inflammatory responses in the aged mice, suggesting that LRRK2 plays an essential role in the regulation of protein homeostasis [26]. Although these molecular and cellular changes are observed only in the kidney but not in the brain of *LRRK2-/- *mice, they bear striking resemblance to processes that are thought to be involved in PD pathogenesis, suggesting that LRRK2 mutations may cause Parkinson's disease and cell death *via *impairment of protein degradation pathways, leading to protein accumulation and aggregation over time. A recent report shows similar gross morphological abnormalities in the kidneys of an independent line of *LRRK2*-/- mice as well as a line of kinase-dead mutant mice of LRRK2 [44]. The presence of similar kidney phenotypes in at least four independent lines of *LRRK2*-/- mice [23,26,44] suggests that this is unlikely an artifact and that LRRK2 play an important role in the cell.

In the current study, we performed an age-dependent analysis of *LRRK2*-/- mice and compared morphological, ultrastructural, and molecular alterations in *LRRK2*-/- mice from 1 month to 20 months of age. We found that gross morphological abnormalities first become evident in *LRRK2*-/- kidneys at 3-4 months of age (Figure 1). Surprisingly, more detailed analysis revealed that the autophagic activity appeared enhanced at young age (*e.g*. 7 months), as evidenced by increased conversion of LC3-I to LC3-II, a reliable marker of autophagosome formation [31], and increased degradation of p62, one of the best characterized autophagy substrates [28], as well as increased degradation of α-synuclein (Figures 3 and 4). By 20 months of age, similar analysis showed reduced autophagic activity in *LRRK2*-/- kidneys (Figures 3 and 4). However, this age-dependent bi-phasic alteration of the autophagic activity is accompanied by progressive accumulation of autolysosomes, reduction of lysosomes, and the ultimate prevalent presence of large lipofuscin granules at 20 months of age (Figure 7).

During the normal process of autophagy (here referred to macroautophagy, the major form of autophagy) [28,31,41], a portion of cytoplasm, including damaged proteins and organelles, is first enclosed by isolation membrane (a double membrane-bound structure) to form an autophagosome, the outer membrane of which then fuses with lysosome to form so-called autolysosome. The internal material, including proteins and lipids, is degraded in the autolysosome by acid hydrolases originated from lysosomes, and the degradation products get recycled back to cytoplasm and are to be used as new building blocks and energy for cellular renovation and homeostasis [28]. Any disruption along this process, such as those that affect initiation and elongation of isolation membrane, autophagosome formation, fusion of autophagosomes and lysosomes, and hydrolytic degradation, would alter the autophagic flux [31]. On the one hand, the presence of a large number of autolysosomes is suggestive of enhanced autophagic flux in *LRRK2*-/- kidneys at young ages (4, 7, and 9-10 months), consistent with increased protein degradation at these ages; On the other hand, the unusual accumulation of such structures may also suggest deficits in turnover and/or recycling of autophagic components, leading to accumulation of autolysosomes, which may evolve into lipofuscin granules through excessive oxidation and crosslinking and eventually result in depletion of autophagic machinery and therefore impaired autophagic activity at old ages (20 months). Deficient regeneration of autophagic lysosomes has been reported to cause accumulation of autolysosomes [45,46]. Consistent with this interpretation, compared with wild-type controls, normal lysosomes were rarely observed in proximal tubules of *LRRK2*-/- kidneys, where there were striking accumulation of autolysosomes (at young ages) and lipofuscin granules (at old ages).

In addition to gross morphological abnormalities observed in *LRRK2*-/- kidneys that first become evident at the age of 3-4 months, the ratio of kidney to body weight in *LRRK2*-/- mice significantly increased at young ages (~10% at 1 month and ~20% at 4 and 7 months) but dramatically decreased at 20 months of age (~50%, Figure 1). We also observed significantly increased levels of lysosomal proteins and proteases (e.g., LAMP-1, cathepsin B, and cathepsin D, including active forms and proforms) in *LRRK2*-/- kidneys beginning as early as one month of age throughout all the ages examined (Figure 6). One possibility is that loss of LRRK2 causes induction of autophagy initially *via *altered kinase signaling. During autophagy induction, synthesis of lysosomal proteins and proteases continues or even up-regulated although other proteins' synthesis is generally down-regulated [28]. At older ages, due to a deficit in clearance or recycling of autolysosomes, the autolysosome-like structures cannot be digested and therefore accumulate and evolve into lipofuscin granules. The increased levels of lysosomal proteins and proteases could be from the accumulated autolysosome-like structures or indigestible lipofuscin granules, both of which contain components originated from lysosomes, including lysosomal proteins and proteases, since the number of lysosomes is not increased, but decreased instead.

**Figure 8:**
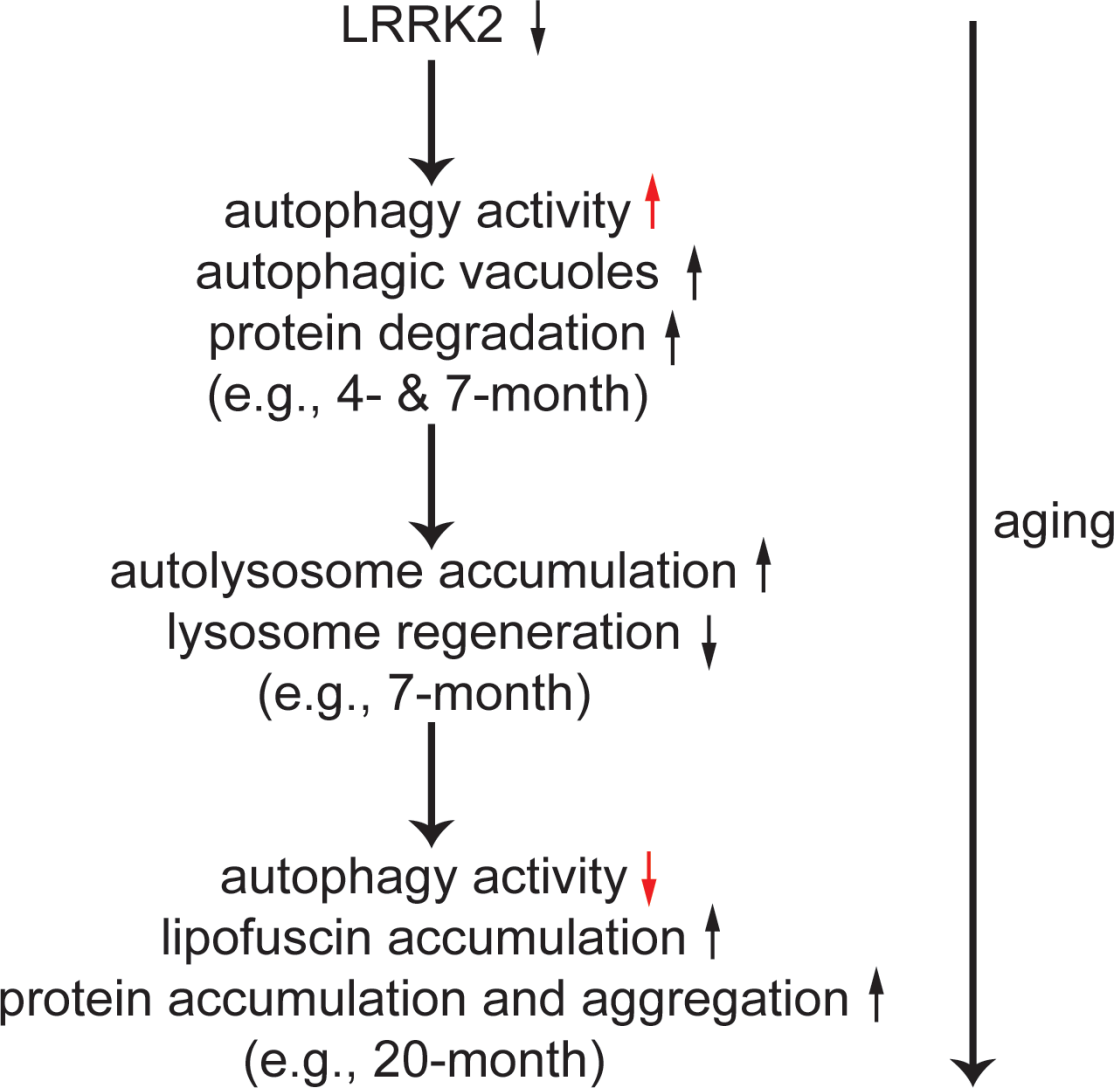
**A schematic model for bi-phasic dysregulation of autophagic activity by loss of LRRK2**. Loss of LRRK2 initially causes induction of autophagy, but later leads to autolysosome accumulation and depletion of the autophagy machinery due to trapping of autophagic components in the form of autolysosomes and the eventual formation of lipofuscin granules, which would in turn result in accumulation and aggregation of a large number of autophagy substrate proteins during aging.

Deficits in autophagy have been implicated in a variety of neurodegenerative diseases with protein aggregation-related pathologies [27,29,47]. Interestingly, increased accumulation of autophagic vacuoles, including both autophagosomes and autolysosomes, has also been reported in postmortem brains of Alzheimer's and Parkinson's disease patients, with likely reasons of either overproduction of autophagic vacuoles or deficit in clearance or recycling of autolysosomes [29,48]. Cathepsin D is also up-regulated in affected neurons. Antibodies to cathepsin D strongly label contents in some of the accumulated autophagic vacuoles, which are identified as autolysosomes, as well as the proteinaceous components of lipofuscins [48]. Our data demonstrate that the autophagy-lysosomal pathway is dysregulated in the absence of LRRK2. Although loss of LRRK2 may initially cause induction of autophagy, deficient clearance or recycling of autophagic components in the absence of LRRK2 would cause trapping of the components of the autophagy pathway in the forms of autolysosomes and the eventual formation of lipofuscin granules due to excessive oxidation and crosslinking and therefore depletion of autophagy machinery (e.g., autophagic lysosomes cannot be reformed.), which would in turn result in accumulation and aggregation of a large number of autophagy substrate proteins during aging (Figure 8). Likely as a consequence or a response to the stresses presumably rendered by the above discussed abnormalities, *LRRK2*-/- kidneys sustain chronic injury, indicated by dramatic and persistent up-regulation of kidney injury molecule-1 (Figure 2), a very sensitive and specific biomarker for epithelial cell injury of proximal renal tubules in various settings [33,34].

Although these molecular and cellular changes are observed only in the kidney but not in the brain of *LRRK2*-/- mice, they are very similar to processes that have been implicated in pathogenesis of PD and other neurodegenerative diseases, making *LRRK2*-/- kidneys a relevant and valuable *in vivo *model, which provides a physiological setting for the studies of LRRK2 function and the identification of the cellular pathways that LRRK2 pathogenic mutations may affect. More questions await further investigation using this unique *LRRK2*-/- kidney as a model. For example, how does loss of LRRK2 cause bi-phasic alteration of autophagic activity? How does loss of LRRK2 lead to autophagy induction as well as deficits in clearance and/or regeneration of autophagy components? Interestingly, it has recently been reported that siRNA knockdown of LRRK2 increases autophagic activity and the R1441C mutation in LRRK2 induces accumulation of autophagic vacuoles of enlarged size in cultured HEK293 cells [49]. Surprisingly, LRRK2 overexpression in cultured HEK293 cells has also been reported to cause autophagy induction through a calcium-dependent pathway [50]. Although these results may seem contradictory with each other, which may be due to the fact that these studies were performed in cell culture systems using immortalized cell lines, rather than an *in vivo *physiological setting, they nevertheless implicate that LRRK2 is important for the dynamic regulation of autophagy function. LRRK2 has also been reported to localize to specific membrane subdomains, including autophagosomes and autolysosomes [49], suggesting that LRRK2 may directly participate in the dynamic process, including formation and clearance, of autophagic vacuoles. What is the role of aging process, which cannot be mimicked in cell culture systems, in this bi-phasic dysregulation of autophagic activity by loss of LRRK2? In addition, LRRK2 has been implicated in both transcriptional and translational regulation [51,52]. Is protein synthesis besides degradation also affected in the absence of LRRK2? Last but not the least, why are these PD-like cellular changes present only in the kidney but not in the brain of *LRRK2*-/- mice? One possibility is that *LRRK2-/- *kidneys suffer the greatest loss of LRRK protein (LRRK1 + LRRK2) because the kidney not only has the highest expression level of *LRRK2 *compared to other organs (5-fold higher than the brain), but also has the least overlapping expression pattern between LRRK2 and LRRK1 [53], the other member of the LRRK family. This may explain why LRRK1 does not compensate for the loss of LRRK2 in the kidney, and loss of LRRK2 causes impairment of the protein degradation pathways and striking age-dependent kidney abnormalities. In the brain, LRRK1 may be able to compensate for the loss of LRRK2. This interpretation is supported by the finding that in the developing brain the expression level of *LRRK1 *is much higher than that of *LRRK2*, and it is broadly expressed [53,54]. We are currently in the process of generating *LRRK1/LRRK2*-deficient mice to determine whether complete loss of LRRK in neurons, especially in dopaminergic neurons where oxidative stress is elevated, results in age-dependent protein aggregation, autophagy alteration, and neurodegeneration. Future studies aimed at addressing these important questions under a physiological setting using our unique *LRRK2*-/- kidney as a model would no doubt help us better understand the normal physiological function of LRRK2 and its role in PD pathogenesis.

## Conclusions

Our latest studies of age-dependent development of phenotypes in *LRRK2-/- *kidneys show that LRRK2 is required for normal regulation of the autophagy-lysosomal pathway. Loss of LRRK2 causes impairment of the protein degradation pathways and striking age-dependent cellular changes in the kidney, which are similar to PD pathogenesis, making the *LRRK2-/- *kidney a unique and valuable model for elucidating the normal physiological role of LRRK2 under its physiological settings. LRRK2 mutations may cause Parkinson's disease and cell death by impairing protein degradation pathways, leading to protein accumulation and aggregation over time.

## Methods

### *LRRK2*-/- mice

The generation and initial characterization of two independent lines (KO1 and KO2) of *LRRK2*-/- mice have been described previously [26]. The mice used in this study were obtained by intercrossing heterozygous littermate mice, which were maintained on B6/129 genetic background. All mouse work follows the protocol approved by Harvard Center for Animal Resources and Comparative Medicine.

### Histological and immunohistochemical analysis

Each mouse was anesthetized by intraperitoneal injection of sodium pentobarbital 15 min after injection of heparin (500 units in saline). The mouse was then transcardially perfused with 20 ml of Ringer's solution containing 0.25 g/L heparin and 5 g/L procaine followed by 25 ml of ice-cold 4% paraformaldehyde in 1× phosphate buffered saline (pH 7.4). The kidneys were dissected out and post-fixed in 4% paraformalhehyde at 4°C overnight and then processed for paraffin embedding following standard procedures. Kidney sections were cut at 8 μm. For immunohistochemical analysis, some tissue sections were subjected to antigen retrieval by microwaving or autoclaving for 10 or 15 min in 10 mM sodium citrate buffer, pH 6.0. Endogenous peroxidase activity was quenched by incubating in 0.3% H_2_O_2 _in methanol. After blocking, sections were incubated with primary antibodies overnight at 4°C, followed by 1-h incubation with biotinylated secondary antibodies and 1-h incubation with Vectastain Elite ABC reagent and then developed using chromogenic DAB substrate (Vector Laboratories). For negative controls, primary antibodies alone or together with secondary antibodies were omitted from the incubation buffer.

### Transmission electron microscopy analysis

Mice were perfused following a procedure similar to that for histological and immunohistochemical analysis above except a mixture of 2.5% paraformaldehyde and 2.5% glutaraldehyde in 0.1 M sodium cacodylate buffer (pH 7.4) was used as the fixative. After overnight post-fixation at 4°C, the dissected tissues were then trimmed to 1-2 mm^3 ^cubes and left in the fixative until processing for embedding in resin. Embedding was performed and ultrathin (60-80 nm) sections were cut by the Harvard Medical School EM facility following a routine protocol. EM images of ultrathin sections were collected on a Tecnai G^2 ^Spirit BioTWIN electron microscope. Some of the tissues from the mice at 9-10 and 20 month of age used for EM analysis were from a third independent line of *LRRK2*-/- mice described previously [23], which also show similar kidney morphological phenotypes.

### Measurement of blood urea nitrogen and serum creatinine

Approximately 0.3 ml of blood was collected from each mouse as described previously [34]. Briefly, mice were anesthetized by intraperitoneal injection of sodium pentobarbital, and blood was collected into heparinized micro-hematocrit capillary tubes by nicking the tail vein of the anesthetized mice near the tip of the tail. Serum was prepared from the collected blood samples and stored at -80°C. Blood urea nitrogen and serum creatinine were measured following the classical methods for renal chemistry as describe previously [34].

### Preparation of RIPA buffer-soluble and -insoluble fractions

Fresh mouse kidneys were homogenized in an ice-cold stringent RIPA buffer (50 mM Tris-HCl, pH 7.4, 150 mM NaCl, 0.1% SDS, 1% Triton X-100, 1% sodium deoxycholate, supplemented with protease inhibitor mixture and phosphatase inhibitor mixtures), followed by sonication. Homogenates were centrifuged at 14,000 × g for 20 min at 4°C to separate supernatants (RIPA buffer-soluble fractions). The resulting pellets were further lysed with a buffer containing 4% SDS and 20 mM HEPES, pH 7.5, supplemented with protease inhibitor mixture and phosphatase inhibitor mixtures by vortexing and sonication, followed by centrifugation at 19,600 × g for 10 min at room temperature to separate the new supernatants (RIPA buffer-insoluble fractions).

### Western blotting and OxyBlot

Equal amount of total proteins from each preparation was loaded and separated in NuPAGE 3-8% Tris-Acetate gels or 4-12% Bis-Tris gels (Invitrogen) and then transferred to nitrocellulose membranes. Oxyblots for detecting protein carbonyls were prepared following the manufacturer's instructions (Millipore). After blocking and overnight incubation with primary antibodies, protein bands of interest were visualized by binding of IRDye-labeled secondary antibodies and band intensity analyzed using Odyssey imaging system (Li-Cor).

### Antibodies

Antibodies used in Western blotting and/or immunohistochemical analyses are: goat pAb anti-mouse Kim-1 (R&D Systems), rabbit pAb anti-ERK1/2 (p44/42 MAP kinase; Cell Signaling), rabbit pAb anti-LC3B (Sigma or Cell Signaling), rabbit pAb anti-GATE-16 (MBL), guinea pig pAb anti-p62 (Progen), rabbit pAb anti-p62 (Wako), rabbit pAb anti-α-synuclein (C-20; Santa Cruz), rabbit pAb anti-LAMP-1 (Cell Signaling), rat mAb anti-LAMP-1 (University of Iowa), rat mAb anti-LAMP-2 (University of Iowa), rabbit pAb anti-cathepsin B (Santa Cruz), rabbit pAb anti-cathepsin D (Calbiochem), rabbit pAb anti-Rab7 (Cell Signaling), mouse mAb anti-GAPDH (Millipore), rabbit pAb anti-β-actin (Cell Signaling), mouse mAb anti-β-actin (Sigma).

### Statistical Analysis

Statistical analysis was performed using Prism 5 (GraphPad Software) and Excel (Microsoft). Data are presented as means ± SEM. Statistical significance was determined by the *P *values of Student t test. Asterisks denote statistical significance (*, *P *< 0.05; **, *P *< 0.01; ***, *P *< 0.001).

## Competing interests

The authors declare that they have no competing interests.

## Authors' contributions

YT conceived and designed the study, carried out experiments for Figures 1, 2, 3, 4, 5, 6, 7, and wrote the manuscript. EG, HY, YL, and HS participated in experiments. TI designed and carried out experiments for Figure 2. HC provided his independently generated *LRRK2*-/- mice (9-10 and 20 months) for EM analysis. JVB participated in experimental design for Figure 2. JS conceived and designed the study, participated in the data interpretation and wrote the manuscript. All authors read and approved the final manuscript.
